# Emergency Bedside Catheter Evacuation of Deep Ganglionic Hematoma Following Recanalization Therapy in Ischemic Stroke

**DOI:** 10.1007/s12028-022-01595-z

**Published:** 2022-09-07

**Authors:** Johann Lambeck, Christine Steiert, Jürgen Bardutzky

**Affiliations:** 1 Department of Neurology and Clinical Neurophysiology, University of Freiburg Medical Center, Breisacherstr. 64, 79106 Freiburg, Germany; 2 Department of Neurosurgery, University of Freiburg Medical Center, Breisacherstr. 64, 79106 Freiburg, Germany

**Keywords:** Secondary hemorrhage thrombolysis, Bleeding complication, Recombinant tissue plasminogen activator, Hematoma thrombectomy rescue technique, Minimally invasive surgery, Intracerebral hemorrhage, Intrahematomal thrombolysis urokinase

The main complication of intravenous application of recombinant tissue plasminogen activator (rtPA) in acute ischemic stroke is secondary intracerebral hemorrhage (sICH), which occurs in 1.7–8.8% of patients [1, 2], typically in the first 48 h following rtPA administration. It is associated with a mortality rate of up to 70%, mainly due to the mass effect of the hematoma [3].

Accordingly, surgical hematoma evacuation appears to be the logical option for potentially achieving a good patient outcome; however, there is currently no evidence-based therapy for sICH after rtPA [4, 5], and many neurosurgeons consider early open surgery dangerous because of frequent bleeding complications, in particular for evacuation of the deep ganglionic region because of its eloquent anatomical localization. The rebleeding risk is further aggravated by reperfusion injury in patients who additionally undergo mechanical recanalization and in patients with tandem occlusion who require platelet inhibition after stenting of the proximal internal carotid artery (ICA). Medical treatment is limited to lowering blood pressure, stabilizing vital signs, and reversal of potential coagulopathy (administration of prothrombin complex concentrate, fresh frozen plasma, or aminocaproic acid) to halt hematoma expansion [4]. However, medical treatment to reverse rtPA-induced coagulopathy is not evidence based and, above all, does not influence the space-occupying effect of the hematoma that is already present. In summary, there are no current evidence-based guidelines that address the medical and surgical management of thrombolysis-associated sICH [4, 5].

A recently published case series described a free-hand bedside catheter technique for emergency hematoma evacuation in well-accessible lobar hemorrhage following systemic thrombolysis within 24 h [3]. We now report a consecutive series of six patients who underwent free-hand bedside catheter evacuation of deep ganglionic hemorrhage into the infarct core after combined systemic thrombolysis and thrombectomy. In this case series, patients were treated with the free-hand catheter technique when the following criteria were fulfilled:

1. Secondary deep ganglionic hemorrhage (sICH) within 48 h of thrombolysis with rtPA (dose [in milligrams] = 0.9 times estimated body weight in kilograms) for treatment of ischemic stroke (with or without mechanical thrombectomy or stenting of the proximal ICA), core infarct volume ≤ 50 mL
2. Space-occupying hematoma volume > 40 mL
3. Reduced level of consciousness due to sICH (somnolence at the least) and deterioration of the NIHSS by at least four points compared to that at admission
4. International normalized ratio (INR) < 1.3, partial thromboplastin time < 35 s, platelet count > 100,000/µL

The study was approved by our local ethics committee (institutional review board registration number 161/19).

Clot evacuation by open craniotomy within 48 h of systemic thrombolysis is not recommended by our institutional protocol. However, the establishment of hemorrhage stability prior to catheter placement is compulsory; hence, all patients received medical treatment. Systolic blood pressure is lowered to < 140 mm Hg using intravenous medication such as urapidil, dihydralazine, and/or clonidine, and oxygen is supplied to patients whose saturation levels are < 95%. Analgosedation and intubation ensue in patients with a Glasgow Coma Score < 9. Four-factor prothrombin complex concentrates are administered in patients with an INR > 1.3 (30–50 IU/kg body weight), and platelets are only transfused when the platelet count is < 100,000/μL (no routine application). The administration of desmopressin for improvement of platelet dysfunction is not recommended by our protocol. Furthermore, the application of aminocaproic acid and fresh frozen plasma is allowed at the discretion of the treating physician.

The locations of the entry point and trajectory of the catheter were determined using a 3D reconstructed computed tomography (CT) scan. The respective distances to the targeted catheter tip location both from the midline and the skin level were determined as previously described [6].

Patients were placed in the supine position for catheter placement. Midline calculation was performed by measuring the distance from one external auditory meatus to the other. Transfer of these coordinates to the patient’s head ensued, a scalp area of 6 × 6 cm was shaved, and the skin was sterilized with ethanol/isopropyl alcohol. Patients received local anesthesia with 5 mL mepivacaine (infiltration of skin and periosteum) and conscious sedation using propofol intravenously (median dose 85 mg, range 50–160 mg). No antibiotic prophylaxis was used.

Twist-drill craniotomy was performed after a 5-mm skin incision. A scaled external ventricular catheter (10F catheter; Spiegelberg, Hamburg, Germany) was inserted at an angle, and the depth was calculated via the 3D reconstructed CT scan. In cases of deep intracerebral hemorrhage occupying the anterior basal ganglia, an anterior trajectory was generally used, with an entry point at the forehead; a posterior trajectory was used in cases of deep-seated intracerebral hemorrhage occupying the posterior basal ganglia or thalamus, with an entry point at the posterior parieto-occipital area.

Mild aspiration with a 10–20-mL hand-held syringe that was connected to the catheter was used to facilitate immediate blood drainage; this was achieved by carefully retracting the piston of the syringe until the first signs of resistance occurred. If no resistance occurred, a new syringe was attached, and aspiration was carefully repeated until the first signs of resistance. At that point, the catheter was attached to the skin and connected to a sterile drainage system. An immediate cranial CT scan was then performed to verify catheter position and residual hematoma volume. The catheter was deemed well positioned when the tip fully engaged the blood clot with its fenestrated segment.

Local thrombolysis was performed as previously described [3], with the following modifications to the inclusion criteria: Only patients with an infarct core of ≤ 50 mL were included. In patients in whom the one-step catheter hematoma aspiration did not significantly reduce the hematoma volume (i.e., to < 50% = surgical target) and did not result in a significant clinical improvement (i.e., > 4 NIHSS points = clinical target), urokinase (5,000 IU, 1 mL) was injected > 3 h after catheter placement, and the system was flushed with 2 mL 0.9% saline. The drain was then clamped for 30 min and reopened for 5.5-h intervals between bouts of local thrombolysis. The pressure threshold for the collecting chamber of the drainage system was set at minus 30–40 cm H_2_O below the foramen of Monroe to allow for gravitational drainage of the lysed clot. Local thrombolysis (5,000 IU urokinase) was repeated every 6 h until hematoma volume decreased to < 50% of the initial volume and the patient showed significant clinical improvement. This treatment regime was limited to a maximum of 4 days; a cranial CT scan was repeated every 24 h to evaluate clot size and ensure that the catheter tip had maintained its intrahematomal position.

Statistical analyses were performed as previously described [3]. Demographic, clinical, and radiological characteristics of the study patients are shown in Table 1. The median patient age was 72 years (interquartile range [IQR]: 68–77). The median event-to-thrombolysis time was 165 min (IQR 142.5–202.5), and the median event-to-thrombectomy time was 290 min (IQR 250–300); all patients had a small infarct core (≤ 12 mL, i.e., below our predefined threshold of 50 mL maximum core). The median thrombolysis-to-clinical deterioration time was 10.5 h (range 6–19), and thrombolysis-to-catheter-time was 12.75 h (range 8.5–21). The catheter was inserted 70 min (range 45–90) after sICH was detected on the CT scan. According to the cranial CT scans, the tip of the catheter had fully engaged the hematoma in all patients so that no replacement procedure was necessary.

**Table 1:** Demographic, clinical, and radiological characteristics of the study patients

	Patient 1	Patient 2	Patient 3	Patient 4^a^	Patient 5	Patient 6^b^
NIHSS at presentation	18	14	16	15	16	15
Transfer from external stroke unit	Yes	Yes	No	No	No	No
Vessel occlusion	M1-branch of the left MCA 90% stenosis of left ICA	M1-branch of the right MCA	M1-branch of the right MCA 90% stenosis of right ICA	M1-branch of the right MCA	M1-branch of the left MCA Occlusion of left ICA	M1-branch of the right MCA
Infarct core at admission	Lenticulostriatal (DWI, 10 mL, external clinic)	Lenticulostriatal (CBV, 5 mL, external clinic)	Striatal (DWI, 12 mL)	None (native CT)	Lenticulostriatal (DWI, 9 mL)	None (native CT)
Symptom onset to thrombolysis time (min)	150	135	240	180	210	140
Symptom onset to recanalization time (min)	300 TICI2b	330 TICI2b	300 TICI2b	240 TICI2b	280 TICI3	225 TICI3
ICA stent acute antithrombotic medication (mg)	Yes aspirin 300 clopidogrel 300	No	Yes aspirin 300 clopidogrel 300	No	Yes aspirin 500 clopidogrel 300	No
Thrombolysis to deterioration time (h)	19	17	10	8	6	11
Thrombolysis to catheter time (h)	21	19.5	12	9.5	8.5	13.5
Deterioration CT to catheter time (min)	55	90	60	45	80	80
NIHSS at deterioration	25	20	24	24	26	23
Ganglionic hematoma volume on CT (mL)	Left 59	Right 47	Right 88	Right 91	Left 96	Right 55
Midline shift (mm)	6	5	8	9	9	3
Aspiration volume (mL)	13	11	14	29	45	19
Postaspiration hematoma volume on CT (mL)	49	38	77	67	57	39
Postaspiration midline shift (mm)	5	4	8	7	6	2.5
NIHSS, post aspiration	22	18	21	22	24	21
Local clot lysis	4 × urokinase 5,000 IU = 24 h	3 × urokinase 5,000 IU = 18 h	6 × urokinase 5,000 IU = 36 h	4 × urokinase 5,000 IU = 24 h	4 × urokinase 5,000 IU = 24 h	4 × urokinase 5,000 IU = 24 h
Hematoma volume after lysis (mL)	13	20	32	12	20	10
Postlysis midline shift (mm)	3	3	5	5.5	4	1
NIHSS at discharge	14 (day 13)	12 (day 14)	13 (day 10)	15 (day 14)	16 (day 11)	14 (day12)
mRS after rehabilitation	3 (week 11)	2 (week 9)	3 (week 8)	4 (week 13)	3 (week 12)	3 (week 10)

CBV, cerebral blood volume, CT, computed tomography, DWI, diffusion weighted imaging, ICA, internal carotid artery, MCA, middle cerebral artery, mRS, modified Rankin scale, NIHSS, National Institutes of Health Stroke Scale, TICI, thrombolysis in cerebral ischemia

^a^Patient 4 received an external ventricular drainage owing to intraventricular extension of hemorrhage and hydrocephalus

^b^Patient 5 received an additional parietal hematoma catheter owing to extension of ganglionic hemorrhage to the parietal cortex (catheter aspiration only without urokinase, aspiration volume 20 mL)

Figure 1 shows the imaging results from a patient with sICH at different time points (on admission, after deterioration, immediately after catheter placement, and after urokinase application at day 3). The median blood volume after deterioration was 74 mL, which significantly decreased by 26% to a median of 53 mL (*p* < 0.05) immediately after catheter aspiration (Fig. 2). Because the hematoma volume had not decreased to < 50% (i.e., predefined surgical target) on the cranial CT scan and clinical improvement following one-step catheterization was not significant (> 4 NIHSS, i.e., predefined clinical target) in any of the patients, local thrombolysis was initiated in all patients > 3 h after catheter positioning. Urokinase was administered for a median duration of 24 h, with a total median dose of 4 × 5,000 IU (range 3–6 × 5,000). The median blood volume thereby further decreased significantly to 16 mL (IQR 12.25–19.75, *p* < 0.05) on the CT scan, 18–36 h after sICH had occurred (24% of initial blood volume). The median midline shift after sICH was 7 mm and decreased to 5.5 mm immediately after catheter aspiration. Following local thrombolysis, a further significant reduction to 3.5 mm (IQR 3–4.75, *p* < 0.05) was observed. The median NIHSS over time was 16 (IQR 15–16) on presentation, 24 (IQR 23.25–24.75) at deterioration, 22 (IQR 21–22) post catheterization, and 14 (IQR 13.25–14.75) at discharge. Following discharge from the rehabilitation clinic (median 12.5 weeks post ictus [IQR 11.25–13.75]), the modified Rankin scale score was 3 (range 2–4) (Table 1). None of the patients died during the study period. Because acute blood samples showed normal values for INR, partial thromboplastin time, and platelet count, none of the patients required prothrombin complex concentrates, platelets, or fresh frozen plasma, and no additional coagulopathy reversal (e.g., with aminocaproic acid) took place. No procedure-related complications, such as rebleeding (as defined by any expansion of hematoma volume after catheter placement or local thrombolysis compared to the previous CT), catheter track bleeding, or infection, were observed.

**Fig. 1:**
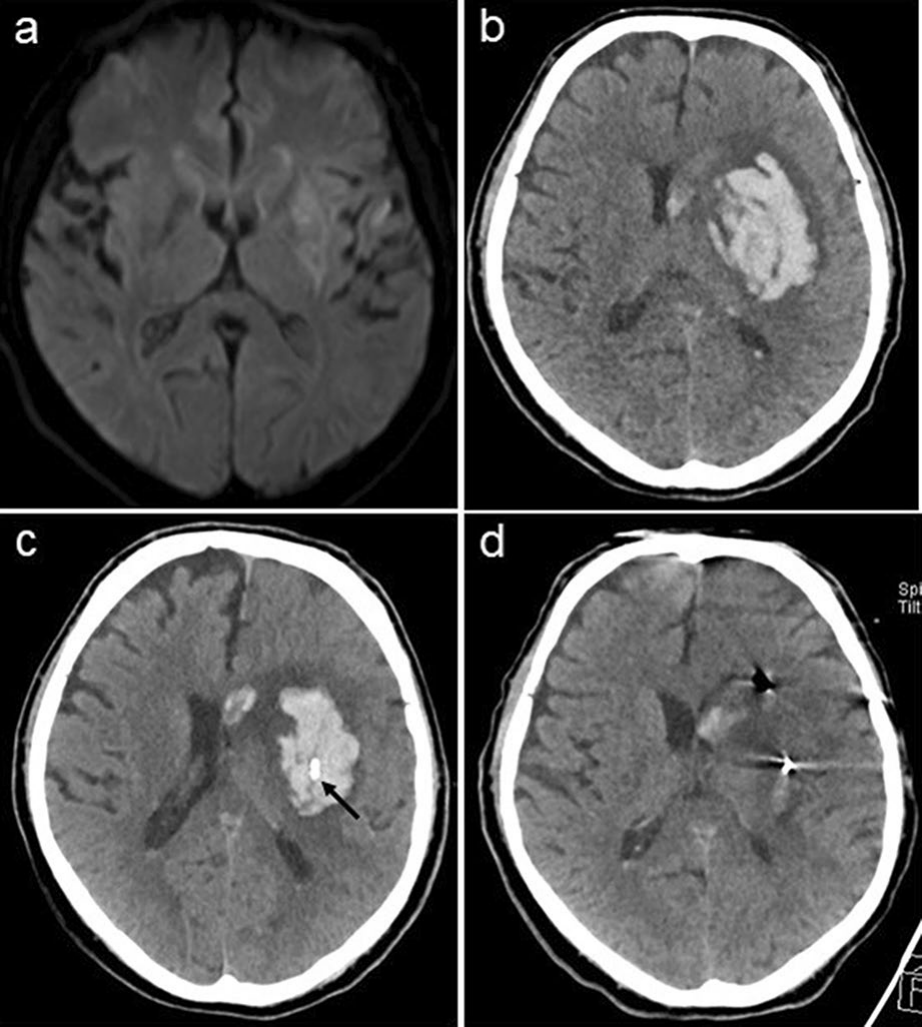
**a** Magnetic resonance (MR) image of patient 1 at the onset of thrombolysis. **b** Computed tomography (CT) image of the same patient following clinical deterioration (NIHSS of 25, with somnolence, global aphasia, severe right-sided hemiparesis, dysarthria) 19 h after thrombolysis. Note the large hematoma in the area of the infarct core (59 mL), with diffuse perifocal edema over the left hemisphere (midline shift, 6 mm). **c** CT image following bedside catheter placement, in which 13 mL of blood was aspirated. The cranial CT image showed good positioning of the catheter (black arrow); however, hematoma size was still substantial (49 mL). The patient’s symptoms had only slightly improved to an NIHSS score of 22; therefore, urokinase administration was initiated. **d** Cranial CT image showing near complete resolution of the hematoma following urokinase administration (5,000 IU) every 6 h for 24 h

**Fig. 2:**
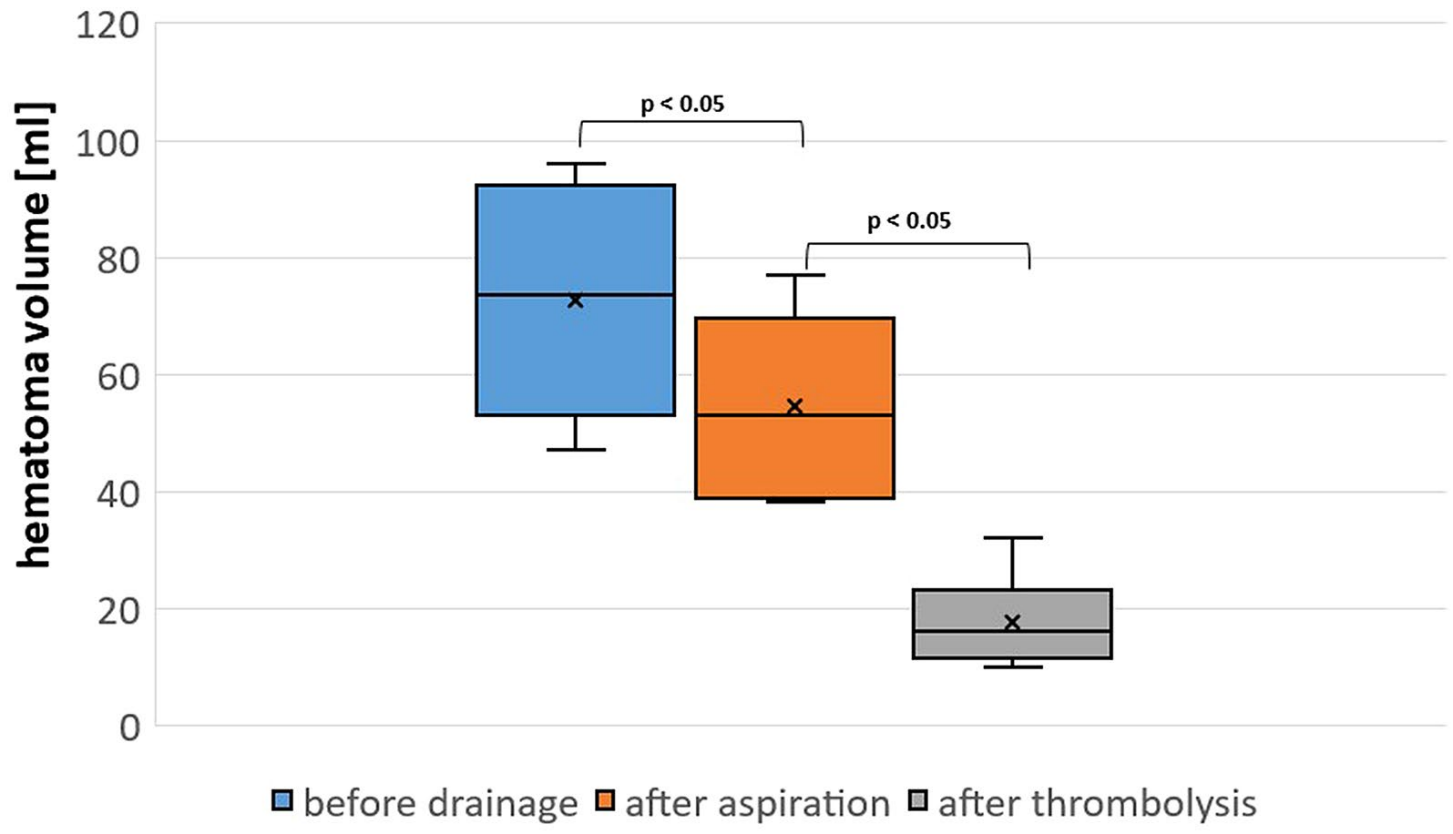
Evolution of hematoma volume before drainage, after catheter aspiration, and after local thrombolysis. Data are shown as median, interquartile range, and range

Recently published data on an emergency bedside catheter evacuation technique in patients with systemic-thrombolysis-associated lobar sICH showed that they are readily accessible to the free-hand technique [3].

As shown in the presented cases, our technique can also successfully be applied in patients with deep ganglionic hemorrhage following recanalization therapy with or without dual antiplatelet therapy. To our knowledge, there are no studies on the use of alternative minimally invasive surgery techniques, such as endoscopic or CT-guided stereotactic evacuation, in this patient cohort. However, these alternative evacuation techniques under vision may be more efficacious in one-step hematoma evacuation. Nevertheless, they do require a more elaborate setting with anesthesia, analgosedation, and intubation; an operating room may not be readily available 24/7.

The advantages of our technique are (1) the short delay from event onset to hematoma evacuation (median 2 h), (2) the short duration of the minimally invasive procedure itself (15 min) without the need for intubation and an operating room, and (3) the relatively fast reduction in hematoma size (26% reduction after one-step aspiration, 76% reduction after 18–36 h of thrombolysis), which is associated with significant mass relief. None of our patients suffered from any catheter-associated side effects.

Furthermore, the first attempt at catheter placement was successful in all patients, with the tip of the catheter fully engaging the hematoma, thus rendering it suitable for local clot lysis. This is most likely due to the large hematoma size, the use of 3D reconstructed CT images for catheter trajectory planning, and the fact that the catheter placement was performed by experienced neurosurgeons.

However, in contrast to the previous report on lobar sICH, the effect of one-step catheterization was less pronounced because hematoma size was only reduced by 26% (versus a 70% reduction in lobar sICH [3]). Nevertheless, all patients showed a slight improvement of 2–3 NIHSS points after catheter aspiration. However, because neither of the abovementioned predefined surgical or clinical targets were met, we initiated additional local thrombolysis in all patients. Local thrombolysis resulted in a further reduction of hematoma size by 76% within 18–36 h of sICH, compared to the initial blood volume. This was associated with a further improvement in NIHSS, which was equal to or better than the NIHSS at admission.

In the MISTIE III trial, a hematoma reduction goal of ≤ 15 mL was targeted and was reached by 58% of the patients. However, a simple transfer to the cohort in our study with larger hematoma volumes (median 73.5 mL as compared to 41.8 mL in the MISTIE III trial) and in which the rebleeding risk is likely to be higher (post rtPA, vulnerable core infarct, definite reperfusion after thrombectomy in all patients with the risk of reperfusion injury, dual antiplatelet therapy in three of six patients after ICA stenting) did not seem rational.

Hence, we postulated that a less strict, more individualized hematoma reduction goal may be better suited to this cohort. It should take the following factors into account: (1) the extent of initial hematoma reduction by catheter aspiration, (2) the subsequent mass-relieving effect, (3) the associated clinical improvement, and (4) the extent of the infarcted brain tissue.

To achieve this, we adopted a hematoma reduction to < 50% as the surgical target and improvement of at least 5 NIHSS points as the clinical target to account for the increased risk of rebleeding in our cohort and allow for a more individualized decision-making.

The major limitations of our case series are the very small sample size, the lack of a control group, the single-center setting, and the nonavailability of urokinase in some countries.

In summary, this is the first report of a free-hand bedside catheter technique for clot evacuation of deep ganglionic sICH, the most feared complication after systemic thrombolysis and mechanical thrombectomy. This procedure is a relatively fast and safe rescue therapy that can be considered in the aforementioned patient cohort in which alternative treatment options are otherwise limited and mortality rate is high.

## Declarations

### Clinical Trial Registration

German Clinical Trials Register (DRKS) identifier DRKS00007908, registered July 15, 2019, https://www.drks.de/drks_web/navigate.do?navigationId=trial.HTML&TRIAL_ID=DRKS00007908
